# A preoperative prediction of lymph node metastasis in early cervical squamous cell cancer with hematologica - based model

**DOI:** 10.7150/jca.85301

**Published:** 2023-06-19

**Authors:** Qiuyuan Huang, Suyu Li, Xiaoying Chen, Xiaohong Liu, Guangrun Zhou, Liyuan Huang, Xiaoyan Li, Kaiwu Lin, Xiangqin Zheng

**Affiliations:** 1 Department of Radiation Oncology, College of Clinical Medicine for Obstetrics & Gynecology and Pediatrics, Fujian Medical University, Fuzhou, China.; 2 Department of Gynecology, College of Clinical Medicine for Obstetrics & Gynecology and Pediatrics, Fujian Medical University, Fuzhou, China.; 3 Department of Radiology, College of Clinical Medicine for Obstetrics & Gynecology and Pediatrics, Fujian Medical University, Fuzhou, China.

**Keywords:** Cervical cancer, Lymph node metastasis, Tumor marker, Nomogram, Squamous cell carcinoma

## Abstract

**Background:** This study aimed to construct a preoperative model predicting lymph node metastasis (LNM) in IB1-IIA2 stage cervical squamous cell cancer (CSCC) based on hematological indexes.

**Merhods:** Between February 2011 and February 2022, 463 patients with IB1-IIA2 stage CSCC underwent radical resection. Patients were allocated to either a model-development cohort (n=337) or a validation cohort (n=126). The final model was determined by comparing different methods of variable selection, and then its discrimination and calibration metrics were evaluated. A predicted probability of LNM < 5% was defined as low risk. ROC curves were used to define high risk.

**Results:** Age, lactate dehydrogenase level, FIGO stage, squamous cell carcinoma antigen, cancer antigen 125, and cancer antigen 199 were identified as critical factors for the construction of the model. The model demonstrated good discrimination and calibration (concordance index, 0.761; 95% confidence interval, 0.666-0.884). In the validation cohort the discrimination accuracy was 0.821 (95% confidence interval, 0.714 - 0.927). In the model-development cohort, 11.9% were classified as low risk with a negative predictive value of 95.0%, and 24.9% were classified as high risk with a positive predictive value of 39.3%.

**Conclusion:** A predictive model was developed and validated for LNM in IB1-IIA2 stage CSCC. The model will assist physicians in appraising the risk of LNM in preoperative patients and could aid in patient counseling and individualized clinical decision-making.

## Introduction

Cervical cancer (CC) is the most common malignant tumor of the female reproductive system, and the fourth most common malignant tumor in women 1. With the benefit of cervical cancer screening programs, early detection of CC has increased worldwide 2. The squamous cell type accounts for > 85% of CC 3. Lymph node metastasis (LNM) is an important prognostic factor in patients with CC 4. The International Federation of Gynecology and Obstetrics (FIGO) clinical staging system was revised in 2018 to include reassessment of lymph node status and tumor size. According to the 2018 FIGO clinical staging system, LNM is classified as stage IIIC, regardless of size or parametrial invasion, and requires concurrent chemoradiotherapy (CCRT). For patients with stage IB1-IIA2 CC, radical hysterectomy with pelvic ± para-aortic lymphadenectomy (RHPL) is considered the standard surgical treatment 5. However, many people who underwent RHPL have been found to suffer from RHPL-related morbidities, including lymphedema, urinary dysfunction, and nerve-site injury, which seriously affect patients' quality of life 6. However, only 10 - 30% of all patients with early stage CC have LNM 7, 8, and cervical squamous cell carcinoma (CSCC) is less likely to progress to LNM than adenocarcinoma 9. This prompts the question of whether CC patients are being subjected to excessive treatment. Therefore, accurate assessment of patients with a risk of LNM is crucial for developing individualized treatment regimens, improving prognosis, and reducing RHPL-related morbidity and mortality 10.

Preoperative assessment of LNM has received much attention in the past decade. Traditionally, assessment of LNM in CC patients is performed using computed tomography (CT) or magnetic resonance imaging (MRI), and determination of metastasis is mainly based on lymph node size 11. However, these are not accurate tools for evaluating LNM 12. Positron emission tomography/computed tomography (PET/CT) scan is more sensitive than CT or MRI alone 12, but it is also more costly and not available everywhere. Because imaging technology is not entirely accurate for evaluating LNM, other indices (e.g., nomograms, risk groups) must be applied to comprehensively judge LNM.

In recent years, nomograms have proven reliable for stratifying LNM risk and prognosis by incorporating valuable factors for oncological outcomes 12-18. Factors found to be particularly valuable for predicting LNM and prognosis include hematological data, age, FIGO stage, and tumor size 14-18. Hematological indices, including neutrophil percentage (NE%), lymphocyte percentage (LY%), hemoglobin level (HB), platelet count (PLT), and squamous cell carcinoma antigen (SCC-Ag), are not only closely related to the prognosis of CC patients 14-16, but also cheaper and more accessible. Other hematological indices are less commonly reported, especially in LNM of CSCC. These measures include white blood cell (WBC) count, lactate dehydrogenase (LDH), cancer antigen 125 (Ca125), cancer antigen 19-9 (Ca19-9), and alpha fetoprotein (AFP).

Therefore, the present study aimed to explore the factors that influence LNM in CSCC patients, evaluate the usefulness of additional hematological indices, and construct a predictive model for LNM in preoperative patients. Additionally, this study aimed to stratify LNM risk based on a predictive model to provide a reference for individualized clinical decision-making in early CSCC.

## Methods

### Study participants

**Figure 1:**
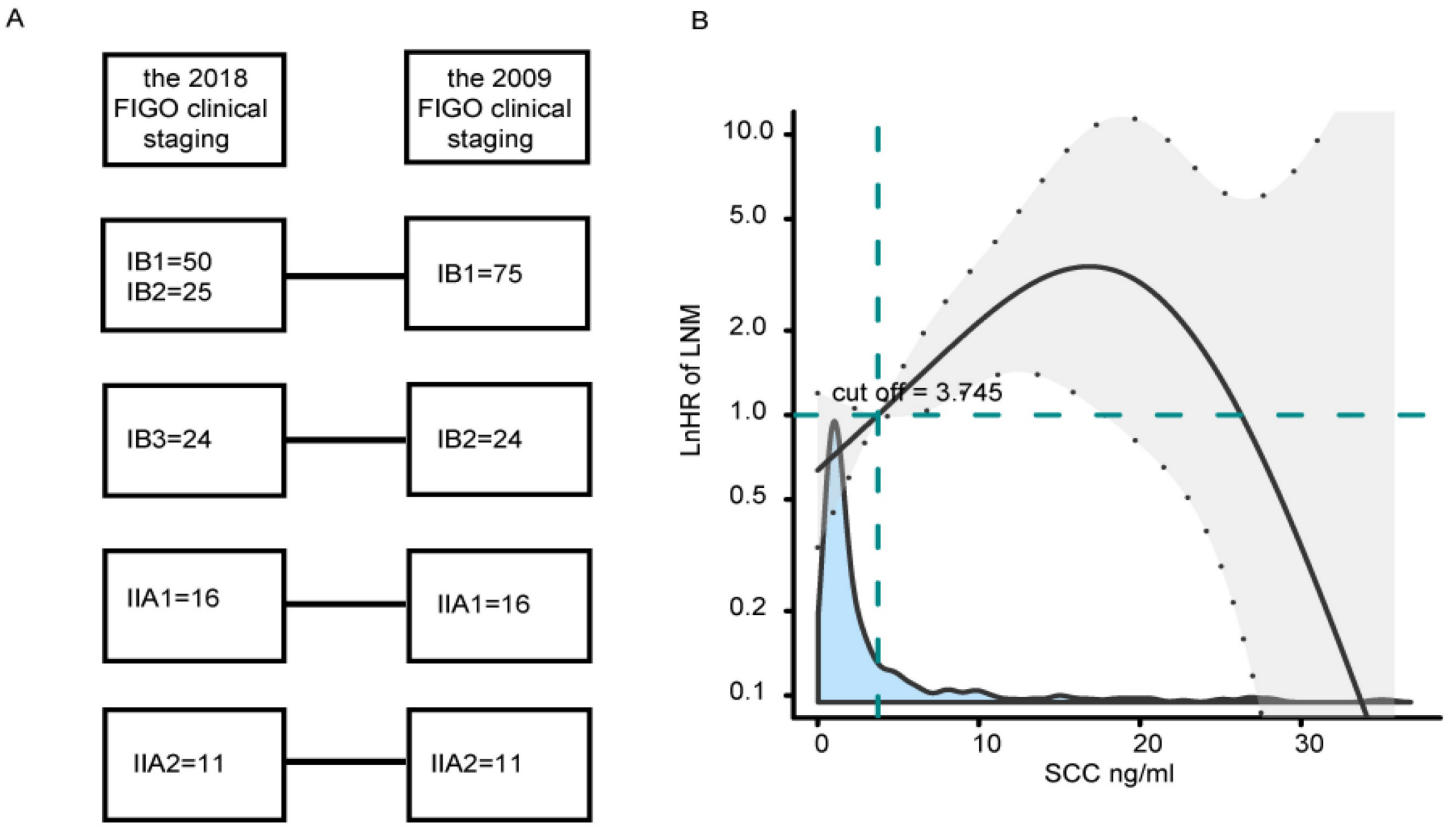
(A) The 2009 FIGO staging compared to the 2018 FIGO clinical staging in a validation cohort. (B) Effects of SCC-Ag on LNM are modeled with a P-spline expansion, with SCC-Ag as a continuous variable. The blue shaded area represents the number of cases. Estimated logarithm hazard ratios (solid lines) with 95% confidence intervals (gray shaded area) for the association of SCC-Ag with LNM according to P-splines smoothing. FIGO, the International Federation of Gynecology and Obstetrics; SCC-Ag, squamous cell carcinoma antigen.

Patients with stage IB1-IIA2 CSCC who underwent surgical treatment at Fujian Maternity and Child Health Hospital between February 2011 and February 2022 were included. Inclusion criteria were as follows: (1) RHPL were performed by the same doctor group; (2) patients were pathologically diagnosed with stage IB1-IIA2 primary CSCC according to the 2009 FIGO clinical staging system; (3) complete clinical data were available, including lymph node dissection; (4) patients did not take antiplatelet agents or receive anticoagulation therapy within 1 month before examination; (5) patients had no history of malignant tumors or immune system diseases. In total, 463 patients satisfied the eligibility criteria. Patients who were diagnosed between February 2011 and October 2018 were assigned to a model-development cohort (n=337), and patients who were diagnosed between November 2018 and February 2022 were assigned to a validation cohort (n=126). In the validation cohort, the 2009 FIGO staging of patients referred to the 2018 FIGO clinical staging (Fig. 1A).

### Data collection

Clinical information, LNM status, preoperative hematological data were collected for all patients. Clinical information included patient age, FIGO stage, tumor size, hypertension, diabetes mellitus, and neoadjuvant chemotherapy (NACT). Pathological features mainly included LNM. According to the surgical pathology of LNM, patients were divided into positive and negative groups. The hematological data were collected one week before treatment and included WBC count, NE%, LY%, HB, hematocrit (HCT), PLT, Ca153, Ca19-9, Ca125, AFP, and SCC-Ag.

The hematological data of the model-development cohort were partially missing (Fig. S1), and the data of the validation group were complete. Multiple imputation 19 was conducted using the mice package in R based on 3 replications. A complete-case analysis was used to assess the sensitivity of the results to bias. All analyses were repeated with the full cohort of data for comparison (Table S1). Age, WBC, NE, LY, HB, HCT, PLT, and LDH, were considered as continuous variables. Other continuous variables (Ca19-9, Ca125, Ca153, AFP) were converted to categorical variables using positive reference ranges. The cut-off values of SCC-Ag were determined by the smooth package in R software 20, 21. Ca153, Ca19-9, Ca125, AFP, SCC-Ag, FIGO stage, hypertension, diabetes mellitus, tumor size, LNM, and NACT were considered as categorical variables (Table S2).

### Statistical analysis

The classification variables were compared using the chi-square test or Fisher's exact probability method. Pearson's chi-squared test was used to test categorical variables of different risk groups.

Variables included in the model were selected in three steps. First, a multivariate and univariate regression analysis was conducted to identify features significantly related to LNM. Second, to avoid over-fitting or under-fitting of the model, three advanced statistical methods, including forward stepwise regression (FSR), best subset regression (BSR) and least absolute shrinkage and selection operator (LASSO), were adopted to select variables in the primary cohort. The criteria for variable selection with FSR and BSR were determined by the Bayesian information criterion (BIC) 22. Cross-validation was used to confirm suitable tuning parameters (λ) for LASSO logistic regression. The most significant features were selected by LASSO 23. Based on results obtained from the univariate and multivariate regression analysis, the FSR, BSR, and LASSO variables were incorporated into the new model. Third, the model was evaluated using receiver operating characteristic (ROC) curves, Harrell's concordance index (C-index), net reclassification index (NRI), and integrated discrimination index (IDI) 24. The final model was selected according to the optimal results of the above analyses.

To assess the fit of the final model, the C-index was used to measure discrimination by calculating the area under the ROC curve, and the Hosmer-Lemeshow test was used to assess calibration. Decision curve analysis (DCA) was used to assess the clinical usefulness of the model and to calculate a net benefit for diverse prediction models at different threshold probabilities by adding the benefits and minimizing the harms 17. The model was evaluated using K-fold cross-validation, which divided the entire data set into six equal segments. The model was fitted to 83.3% of the data (training set) and tested on 16.7% of the data (test set), a process that was repeated six times 25. For external validation, the model was applied on a validation cohort. Using the same methods described above, the discrimination and calibration of the model were tested. as defined in the previous article, a predicted probability of LNM<5% was defined as low risk 17. ROC curve was used to determine a specific cutoff value of high risk.

## Results

### Clinical characteristics

A total of 463 patients were included in this study. Patients diagnosed between February 2011 and October 2018 were assigned to a model-development cohort (n = 337) and patients diagnosed between November 2018 and February 2022 were assigned to a validation cohort (n = 126). Characteristics of the model-development and validation cohorts are shown in Table 1. LNM frequencies for the model-development and validation cohorts were 15.4% and 19.0%, respectively (*p* = 0.35).

### Value-dependent effects of SCC-Ag on LNM

To quantify the effect and optimal cut-off value of SCC-Ag on LNM, a multivariate logistic regression analysis was conducted using P-splines in smoothHR of the R software. The analysis indicated that the risk of LNM increased sharply when SCC-Ag was greater than 3.75 ng/ml (Fig. 1B). These findings confirmed an SCC-Ag-dependent effect, and an SCC-Ag cutoff of 3.75 ng/ml was used to stratify patients into two sub-groups depending on whether their SCC-Ag value was less than or greater than the cutoff.

### Univariate and multivariate analysis for LNM

The univariate analysis indicated that SCC-Ag, Ca125, Ca19-9, HCT, FIGO stage, and LDH were all linked to LNM. Multivariate analysis confirmed the following as independent factors for LNM: SCC-Ag, Ca19-9, and Ca125. The findings of the univariate and multivariate logistic regression analysis are shown in Table S3 (all *P* < 0.05).

### Variable Selection

**Figure 2:**
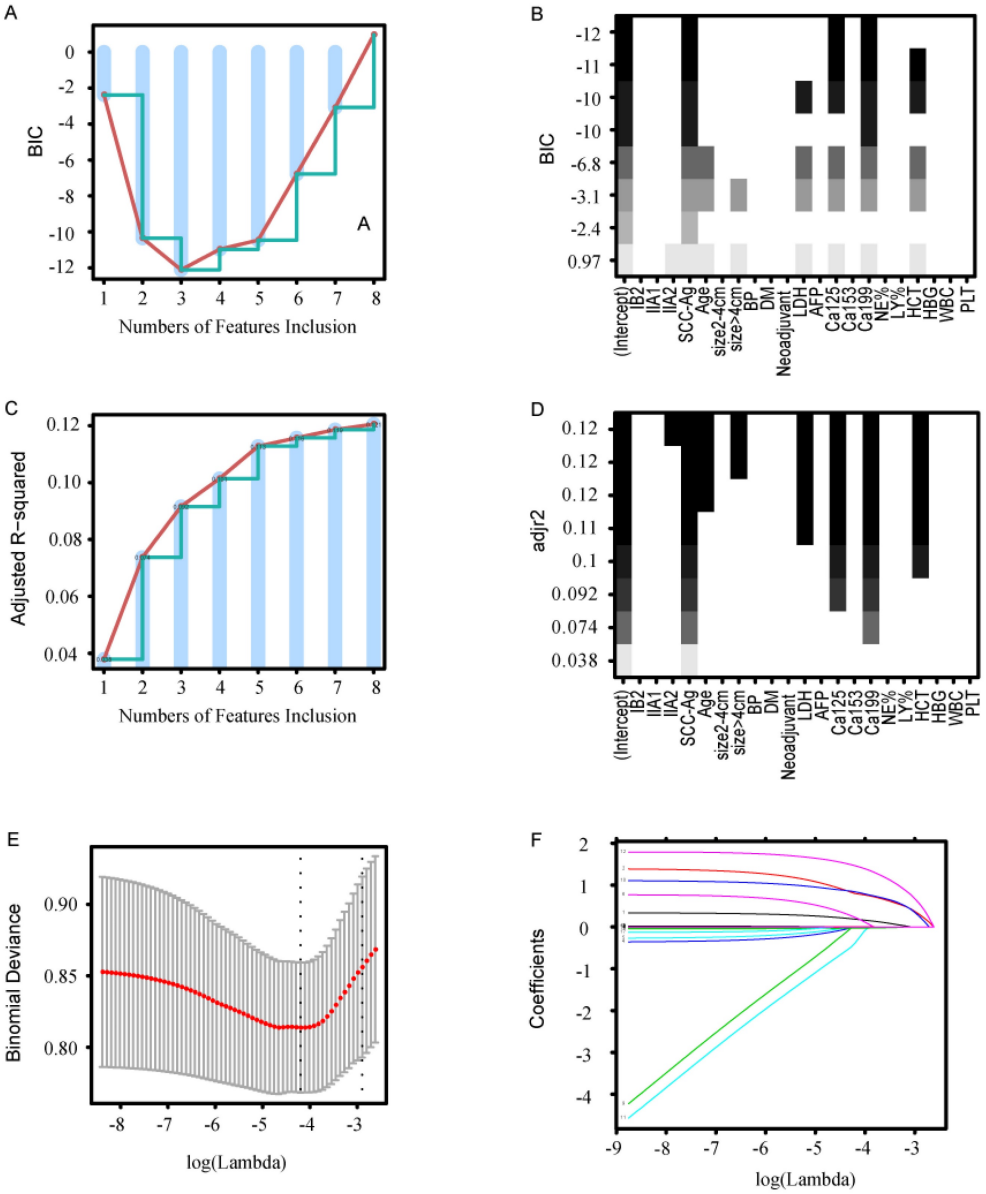
Methods of variable selection. (A, B) Variable selection using the FSR method. (A) The minimum BIC was -12.1 when there was an inflection point in the broken line. (B) The ordinate represents BIC, and the abscissa represents the variable. (C, D) Variable selection using the BSR method. (C) The maximum adjusted R-squared was 0.121 when there was an inflection point in the broken line. (D)The ordinate represents adjusted R-squared, and the abscissa represents the variable. (E) Cross-validation was applied for tuning parameter (λ) selection. (F) The LASSO coefficient profile of LNM-related variables in the model-development cohort. BIC, Bayesian information criterion; FSR, forward stepwise regression; BSR, best subsets regression; LASSO, least absolute shrinkage and selection operator; LDH, lactate dehydrogenase; Ca19-9, cancer antigen 199; Ca125, cancer antigen 125; LY%, lymphocyte percentage (LY%); HCT, hematocrit.

Three common methods (FSR, BSR, and LASSO) were used to select variables. FSR yielded three variables: SCC-Ag, Ca19-9, and Ca125. The minimum BIC was -12.1 because there was an inflection point in the broken line (Fig. 2A, B). BSR yielded eight variables: FIGO, age, tumor size, LDH, CA125, HCT, Ca19-9, and SCC-Ag. The maximum adjusted R-squared value was 0.121 (Fig. 2C, D). All selected variables had significant statistical difference (all *P* < 0.05). LASSO yielded eight statistically significant variables: FIGO, age, tumor size, LDH, HCT, Ca19-9, Ca125, and SCC-Ag (lambda value 0.015). As shown in Figure 2E, F, a coefficient profile figure was produced against the ln (λ) sequence.

### Development of the final model

**Figure 3:**
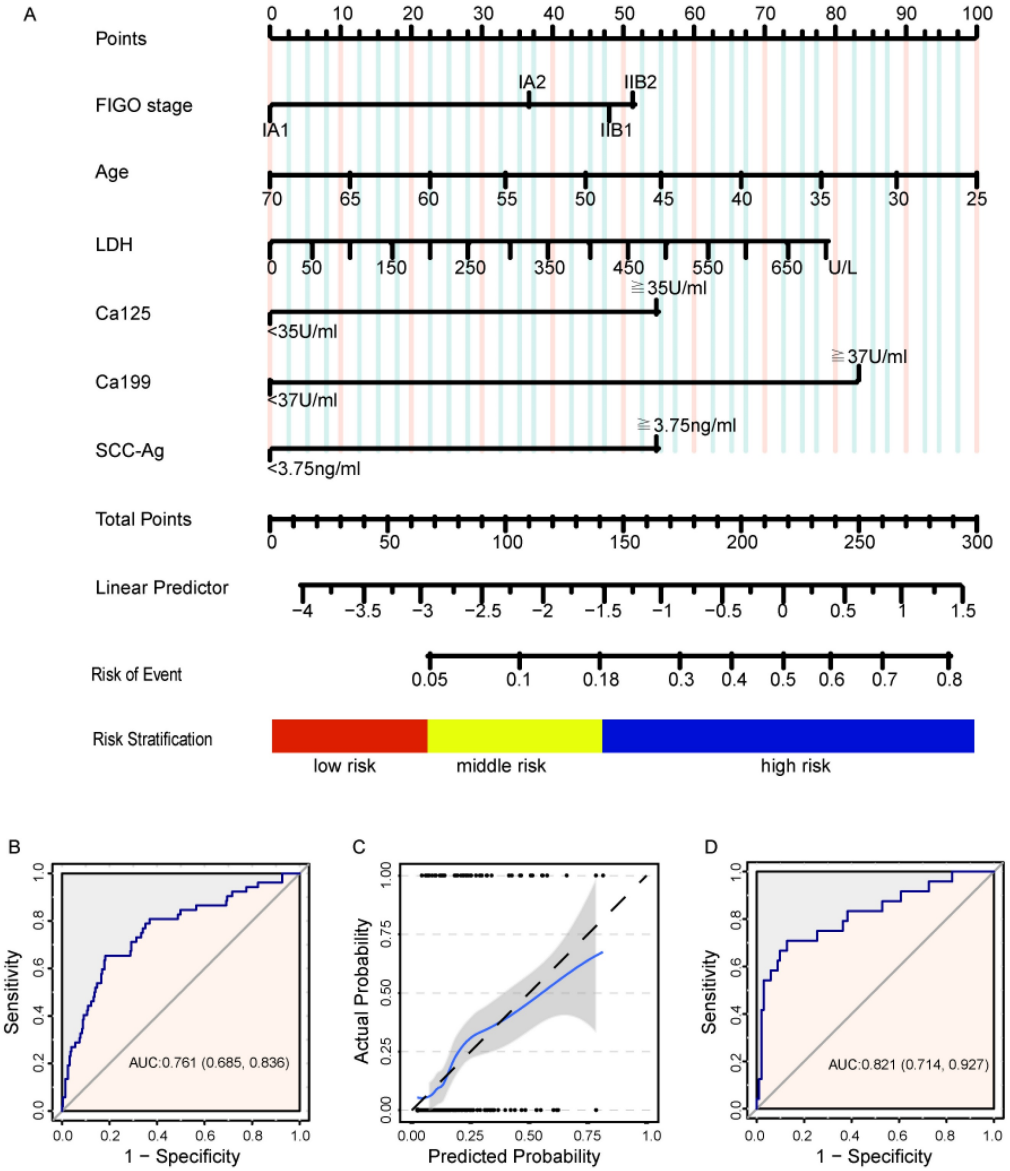
(A) A nomogram predicting LNM in IB1-IIA2 stage cervical squamous cell cancer. A total score of 66.7 was assigned a value of 0.05 and defined as low risk for LNM. A total score of 140.1 was assigned a value of 0.18 and defined as high risk for LNM. (B) Discrimination plots for the model-development cohort. (C) The solid 45-degree line represents the ideal prediction, and the broken line shows the observed results of the model. A good prediction model is one that has a close fit to the solid line. (D) Discrimination plots for the validation cohort.

Based on the results of the univariate and multivariate regression analysis, the FSR, BSR, and LASSO, six variables were incorporated into model1: FIGO, age, LDH, SCC-Ag, Ca19-9, and Ca125 (Fig. 3A). Model2, model3, and model4 were built separately according to the results of FSR, BSR, and LASSO. The choice of the final model was determined by ROC curve, C-index, and NRI and IDI, which were also used to evaluate the efficiency of the models (Table 3 and Fig. S2A-C).

Model2 showed the smallest C-index (0.670, 95% CI: 0.593, 0.746) among the four models, and fit most poorly (*P* < 0.05). Model1, model3, and model4 had similar discriminative ability, but model3 and model4 used more variables (*P* > 0.05). Using an appropriate number of variables, model1 showed good discrimination. Therefore, model1 was used for the final model.

### Model performance

Suitable calibration was verified for the model development and validation cohorts. The calculation of points and linear predictors is shown in Table S4. In the development cohort, the discrimination accuracy of the model was 0.775 (95% CI: 0.666, 0.884; Fig. 3A, B). A good prediction model would have a close fit to the dashed line (Fig. 3C). Brier Scores closer to 0 indicate better calibration. The Hosmer-Lemeshow test indicated a satisfactory fit for the model (Brier = 0.114, *X*^2^ = 4.765, *P* = 0.782), and a DCA of the model is shown in Figure S3A. Internal and external verification of the model involved discrimination and calibration. Internal verification is shown in Supplemental Figure 3B (C-index = 0.761, Brier = 0.112, *X*^2^ = 2.554, *P* = 0.388). External verification is shown in Figure 3C and Figure S3C (C-index = 0.821, Brier = 0.125, X2 = 11.703, *P* = 0.165).

### LNM risk groups

A predicted probability of LNM < 5% was defined as low risk. ROC curves confirmed this to be an optimal cut-off (Fig. S4). Using linear-predictors of the model, a predicted probability of LNM > 18% was defined as high risk (Fig. 3A). In a multivariate analysis, the risk groups were confirmed as the most important predictors (Table S5). In the model-development cohort, 11.9% were classified as low risk, with a negative predictive value (NPV) of 95.0%; 24.9% were classified as high risk, with a positive predictive value (PPV) of 39.3%. In the validation cohort, 19.8% were identified as low risk, with a NPV of 96.0%, and 15.1% were identified as high risk, with a PPV of 68.4% (Fig. 3A). Table S6 shows the three patients with LNM falsely identified as low risk. Table 3 shows the different LNM risk groups in the model-development and validation cohorts.

## Discussion

The present study identified FIGO, age, LDH, SCC-Ag, and Ca19-9 as significant variables for predicting LNM in CSCC. Notably, this is the first known report of LDH and Ca19-9 being used in this context. The predictive model was developed and validated using both internal and external verification. It demonstrated good discrimination and calibration (C-index = 0.775, Brier = 0.114, *P* = 0.782). In the validation cohort, the discrimination accuracy was 0.821 (Brier = 0.125, *P* = 0.165). The predictive model correctly stratified LNM risk: the rate of was remarkably low in the low-risk group and relatively high in the high-risk group. The model provides a clinical reference for the individualized treatment of patients with early stage CSCC.

LNM is an important prognostic factor in patients with CC 5. In this study, the LNM frequencies for the model-development and validation cohorts were 15.4% and 19.0%, respectively (*P* = 0.35). This finding is consistent with that of other investigators who reported that 10 - 30% of patients with early-stage CC have LNM 7, 8.

Before structuring the model it was necessary to determine whether the hematological data should be expressed as continuous or categorical variables and, if categorical, which cutoff values to use. In the univariate model, all variables were initially considered as both continuous and categorical. From this analysis it became clear that Ca19-9, Ca125, and SCC-Ag were more meaningful as continuous variables, and LDH, NE, LY, and HCT were more meaningful as categorical variables (Fig. S5). Some studies have shown that hematological indices, including NE, LY, HB, PLT, and SCC-Ag, are closely related to LNM risk and prognosis of CC patients 14-16. The cutoff values of hematological indices were selected based on ROC curve and positive reference values of the instruments. In the present univariate and multivariate analyses, Ca153, Ca19-9, Ca125, and AFP were significantly related to LNM risk, but SCC-Ag was not. P-splines smooth was used to determine the optimal cutoff values 21 because it is based on a non-linear relationship between the continuous variable and LNM 20, 26. The risk of LNM declined sharply when SCC-Ag was greater than 20 ng/mmol, but there were fewer patients in this range, so this cutoff offers only low specificity. The risk of LNM increased sharply for SCC-Ag values greater than 3.75 ng/mmol, so this cutoff was selected as an optimal cutoff value for LNM.

In order to ensure the reliability of the model, four variable selection methods were used to identify possible combinations of potential predictors 22, 23. The efficiency and discrimination of each model were examined by ROC curve, C-index, NRI, and IDI. FIGO, age, SCC-Ag, LDH, Ca125, and Ca19-9 were incorporated into the final model. This was in good agreement with previous studies that incorporated FIGO, age, and SCC-Ag into the nomogram. Those studies showed that young age, late stage, and elevated levels of Ca125, Ca19-9, and SCC-Ag were significantly associated with LNM in CC, as well as poor prognosis 17, 18, 27. In addition, elevated LDH was predictive of an aggressive phenotype, heavy tumor burden, and higher likelihood of lymphovascular space invasion (LVSI), LNM, and poor prognosis 28. In contradiction to earlier findings, this is the first study, to our knowledge, that investigates the predictive model of LNM in CC using LDH and Ca19-9. It is important that variables be selected with care to combine tumor markers and LDH.

For both internal and external validation, the model was evaluated in terms of its discrimination and calibration. The original model showed good measures of discrimination and calibration (C-index = 0.775, Brier = 0.114, *X^2^* = 4.765, *P* = 0.782). Overall similarities were noted in the internal verification (C-index = 0.761, Brier = 0.112, *X^2^* = 2.554, *P* = 0.388). For external validation, data were collected from November 2018 to February 2022. The FIGO clinical staging system was revised in 2018 to include reassessment of lymph node status and tumor size by imaging technology rather than physical examination 5, 11. The 2009 FIGO clinical staging of patients was compared to the 2018 FIGO clinical staging in a validation cohort (Fig. S1). Compared with the model-development cohort, discrimination was slightly better and model-fit slightly poorer in the validation cohort (C-index=0.821, Brier=0.125, *X^2^* = 11.703, *P* = 0.165). There are several possible reasons for this deviation. First, with the updated FIGO clinical staging, imaging technology rather than physical examination might have provided better differentiation and efficiency as compared to physical examination. Second, there was a significant difference in median age between the model-development and validation cohorts (47.0 vs. 50.0, *P* = 0.011). Other variables included in the model had no significant differences between cohorts (all *P* > 0.05). Age showed borderline significance for LNM in the multivariate analysis (*P* = 0.059). This is likely due to increased screening for CC in older women in the past decade 29 and might result in poor model fit. With regard to internal and external validation, the model showed good discrimination and calibration for the 2009 FIGO staging criteria, and extrapolation to the 2018 FIGO staging criteria might be reasonably approximated.

Patients were classified based on different risk levels for LNM. In the multivariate analysis, risk group was confirmed as the most important predictor. The predicted probability for a low-risk group was < 5%, with a NPV greater than 95.0%. The predicted probability for a low-risk group with > 18% risk yielded a PPV of 39.3% in model-development cohorts. The PPV was even higher (68.4%) in validation cohorts. This suggests that the model is also applicable to the 2018 FIGO staging system, and that imaging technology rather than physical examination provided more accurate information for staging 5. Notably, a total of three patients were incorrectly classified as low risk by our model. Two patients had LVSI or deep stromal invasion upon final pathologic examination. LVSI and deep stromal invasion could not be assessed in the majority of preoperative patients because most patients underwent punch biopsies; only a minority of patients underwent conization biopsies. One patient was incorrectly classified as low risk because of discrepancies between the 2009 and 2018 FIGO staging systems. The patient was initially classified as IB1 stage with endogenous tumor (size > 4cm), but according to 2018 FIGO staging the patient would have been classified as IB3 stage (equivalent to 2009 FIGO stage IB2). LVSI, deep stromal invasion, and tumor size are all known to be powerful predictors of LNM in CC patients 30. It has also been reported that preoperative prediction of LNM in CC can be improved by including radiomics in the nomogram 12, 17. The risk of LNM may be more accurately predicted by incorporating radiomics into the preoperative nomogram, especially for the endogenous tumor.

The most important benefit of the present model is that it allows for preoperative risk assessment using hematological indices. This makes it a useful tool for individualized clinical decision-making by physicians and patients. Theoretically, after complete LN removal, patients who are truly negative for LNM would not benefit from RHPL. RHPL can result in morbidities such as vessel injuries, nerve injuries, infection, lymphocysts, and lymphedema, and should not be performed unless necessary 6. Recent data suggest that sentinel LN (SLN) biopsy could be established as a method of LN staging in patients with early CC, but SLN biopsy is not routinely performed 31. After combining the model-development and validation cohorts, the NPV of the low-risk group was greater than 95.0%. Therefore, the model could provide a reference for when SLN biopsy should be performed instead of RHPL. Furthermore, the model also provided a basis that it was necessary to comprehensively assess lymph node status in the high-risk group. For example, PET/CT, SLN biopsy, SLN imaging, and especially ultrasound-guided fine needle aspiration cytology offered better sensitivity and specificity 31, 32. Patients with LNM are assigned to stage IIIC, for which CCRT is the standard treatment 5. Comprehensive assessment of lymph node status would help to avoid the double blow of surgery and CCRT. Overall, the model described here may help clinicians to design individualized treatment for patients with early stage CSCC.

There were some limitations in this study. First, the data were of limited size, retrospective, and derived from a single institution, introducing the potential for selection and confounding biases. Prospective, multicenter studies are still required to confirm the predictive value of the model in a clinical practice environment. Second, because of the difference in age and the updating of FIGO stages in 2018, discrimination and calibration metrics were slightly different across the validation cohorts. Furthermore, a total of three patients were incorrectly classified as low risk. The performance index of the model may increase by incorporating radiomics, punch biopsies, and other indices (such as detection of human papilloma virus) into the nomogram.

## Conclusion

In conclusion, a preoperative model based on hematological indices was developed and validated to predict LNM in IB1-IIA2-staged CSCC patients. The predictive model was able to accurately stratify LNM risk. Based on model output, clinicians may be assisted in decisions such as whether to perform RHPL in low-risk patients, or to pursue more aggressive examination and treatment in high-risk patients. Overall, the model promises to help clinicians strategize individualized treatment for patients with early stage CSCC.

## Supplementary Material

Supplementary figures and tables.

## Figures and Tables

**Table 1:** Characteristics of the model-development and validation cohorts

Characteristics	Total (n = 463)	the develop-model cohort (n = 337)	the validation cohort (n = 126)	*P*	statistic
FIGO, n (%)				0.062	7.347
IB1	237 (51.2)	162 (48.1)	75 (59.5)		
IB2	86 (18.6)	62 (18.4)	24 (19)		
IIA1	73 (15.8)	57 (16.9)	16 (12.7)		
IIA2	67 (14.5)	56 (16.6)	11 (8.7)		
Age, Median (IQR)	48.0 (42.0, 54.0)	47.0 (42.0, 53.0)	50.0 (43.0, 58.0)	0.011	6.396
LNM, n (%)				0.35	0.875
negative	387 (83.6)	285 (84.6)	102 (81)		
positive	76 (16.4)	52 (15.4)	24 (19)		
LDH,Median (IQR)	165.9(141.8, 203.1)	162.4 (138.0, 208.8)	169.9 (150.2, 200.0)	0.284	1.147
Ca125, n (%)				0.595	0.283
< 35U/ml	431 (93.1)	315 (93.5)	116 (92.1)		
≥ 35U/ml	32 (6.9)	22 (6.5)	10 (7.9)		
Ca19-9, n (%)				0.776	Fisher
< 37U/ml	447 (96.5)	326 (96.7)	121 (96)		
≥ 37U/ml	16 (3.5)	11 (3.3)	5 (4)		
SCC-Ag, n (%)				0.513	0.427
< 3.75ng/ml	348 (75.2)	256 (76)	92 (73)		
≥ 3.75ng/m	115 (24.8)	81 (24)	34 (27)		

**Table 2:** Effectiveness of the four models

	C-index (95% CI)	△C-index (P value)	NRI (95% CI)	△NRI (P value)	IDI (95% CI)	△IDI (P value)
Model1	0.761 (0.685, 0.836)	-	-		-	
Model2	0.670 (0.593, 0.746)	0.004	-0.144 (-0.291, 0.004)	0.057	-0.0407 (-0.068, -0.0135)	0.003
Model3	0.767 (0.694, 0.839)	0.694	0.0018 (-0.079, 0.083)	0.965	0.022 (0.002 ,0.04)	0.169
Model4	0.741 (0.664, 0.819)	0.381	-0.072 (-0.168, 0.024)	0.143	0.011 (-0.015, 0.037)	0.399

Model1: FIGO, age, LDH, Ca19-9, Ca125, SCC-Ag Model2, with variables selected by FSR: Ca19-9, Ca125, SCC-Ag Model3, with variables selected by BSR: FIGO, age, tumor size, LDH, HCT, Ca19-9, Ca125, SCC-Ag Model4, with variables selected by LASSO: FIGO, age, diabetes mellitus, LY%, HCT, Ca19-9, Ca125, SCC-Ag

**Table 3:** LNM risk groups in the model-development and validation cohorts.

Characteristics	the model-development cohort			the validation cohort	
	negative	positive		negative	positive
Total	285 (84.6)	52 (15.4)		102 (81.0)	24 (19.0)
low risk group	38 (95)	2 (5)		24 (96)	1 (4)
middle risk group	194 (92.4)	16 (7.6)		72 (87.8)	10 (12.2)
high risk group	53 (60.9)	34 (39.1)		6 (31.6)	13 (68.4)
