# Supplementary material for: A preoperative prediction of lymph node metastasis in early cervical squamous cell cancer with hematologica - based model
Source: J Cancer. 2023 Jun 19;14(10):1763–72. doi: 10.7150/jca.85301 (PMC10355196; doi:10.7150/jca.85301)
Supplement: Supplementary file 1 — Supplementary figures and tables. [file jcav14p1763s1.pdf]

Supplementary Information

Table S1. Sensitivity analyses of pre- and post- multiple imputation.

Characteristics	pre		post	
Variable	OR(95% CI)	*P* value	OR(95% CI)	*P* value
(Intercept)	1.21 (0.11 ~ 13.31)	0.877	1.25 (0.12 ~ 13.47)	0.856
HBG	0.98 (0.96 ~ 1)	0.123	0.98 (0.96 ~ 1)	0.11
NE%	1.02 (1 ~ 1.05)	0.091	1.02 (1 ~ 1.05)	0.089
WBC	1.02 (0.88 ~ 1.19)	0.772	1.02 (0.88 ~ 1.18)	0.788
LY%	0.97 (0.94 ~ 1)	0.064	0.97 (0.94 ~ 1)	0.069
PLT	1 (1 ~ 1)	0.858	1 (1 ~ 1)	0.863
HCT	0.94 (0.88 ~ 0.99)	0.024	0.94 (0.88 ~ 0.99)	0.022
LDH	1 (1 ~ 1)	0.024	1 (1 ~ 1)	0.02
AFP	0.94 (0.8 ~ 1.1)	0.431	0.94 (0.81 ~ 1.1)	0.47
Ca125	1.01 (1 ~ 1.02)	0.101	1.01 (1 ~ 1.02)	0.11
Ca153	1 (0.96 ~ 1.04)	0.977	1 (0.96 ~ 1.04)	0.987
Ca19-9	1.02 (1 ~ 1.04)	0.047	1.02 (1 ~ 1.04)	0.046

Table S2. Patient characteristics by LNM in the model-development cohort.

Characteristics	Total (n = 337)	negative(n = 285)	positive (n = 52)	*p*	statistic
FIGO, n (%)				0.025	9.354
IB1	162 (48.1)	147 (51.6)	15 (28.8)		
IB2	62 (18.4)	48 (16.8)	14 (26.9)		
IIA1	57 (16.9)	46 (16.1)	11 (21.2)		
IIA2	56 (16.6)	44 (15.4)	12 (23.1)		
Age, Median (IQR)	47.0 (42.0, 53.0)	48.0 (42.0, 53.0)	45.0 (39.0, 52.0)	0.072	3.242
LDH, Median (IQR)	162.4 (138.0, 208.8)	160.5 (136.0, 193.5)	183.2 (149.0, 333.0)	0.006	7.685
NE%, Median (IQR)	57.9 (51.7, 64.3)	57.5 (51.7, 63.8)	60.6 (52.5, 67.2)	0.084	2.986
WBC, Median (IQR)	5.9 (4.8, 7.1)	5.9 (4.9, 7.1)	6.1 (4.8, 7.3)	0.938	0.006
LY%, Median (IQR)	31.2 (26.2, 38.0)	31.6 (27.0, 38.1)	29.9 (24.4, 35.7)	0.104	2.641
PLT, Median (IQR)	250.0 (205.0, 300.0)	249.0 (204.0, 298.0)	253.0 (208.8, 305.8)	0.735	0.114
HCT, Median (IQR)	36.3 (33.4, 38.3)	36.5 (33.7, 38.5)	34.9 (31.6, 37.1)	0.007	7.158
Tumor size, n (%)				0.746	0.585
< 2cm	132 (39.2)	114 (40)	18 (34.6)		

2 – 4cm	118 (35.0)	99 (34.7)	19 (36.5)		
> 4cm	87 (25.8)	72 (25.3)	15 (28.8)		
BP, n (%)				0.274	1.194
No	266 (78.9)	222 (77.9)	44 (84.6)		
Yes	71 (21.1)	63 (22.1)	8 (15.4)		
DM, n (%)				0.261	Fisher
No	311 (92.3)	265 (93)	46 (88.5)		
Yes	26 (7.7)	20 (7)	6 (11.5)		
Neoadjuvant, n (%)				0.319	0.993
No	215 (63.8)	185 (64.9)	30 (57.7)		
Yes	122 (36.2)	100 (35.1)	22 (42.3)		
SCC-Ag, n (%)				0.194	1.687
< 3.75ng/ml	164 (48.7)	143 (50.2)	21 (40.4)		
≥ 3.75ng/ml	173 (51.3)	142 (49.8)	31 (59.6)		
AFP, n (%)				1	Fisher
< 8.78ng/ml	333 (98.8)	281 (98.6)	52 (100)		
≥ 8.78ng/ml	4 (1.2)	4 (1.4)	0 (0)		
Ca125, n (%)				0.003	Fisher
< 35U/ml	315 (93.5)	272 (95.4)	43 (82.7)		
≥ 35U/ml	22 (6.5)	13 (4.6)	9 (17.3)		
Ca153, n (%)				1	Fisher
< 31U/ml	333 (98.8)	281 (98.6)	52 (100)		
≥ 31U/ml	4 (1.2)	4 (1.4)	0 (0)		
Ca19-9, n (%)				0.003	Fisher
< 37U/ml	326 (96.7)	280 (98.2)	46 (88.5)		
≥ 37U/ml	11 (3.3)	5 (1.8)	6 (11.5)		

Table S3. Univariate and multivariate logistic regression for predicting LNM.

Characteristics	Univariate analysis		Multivariate analysis	
	OR(95% CI)	*P* value	OR(95% CI)	*P* value
FIGO IB2	2.86 (1.29~6.35)	0.01	2.19 (0.88~5.47)	0.092
FIGO IIA1	2.34 (1.01~5.46)	0.048	2.73 (0.99~7.8)	0.051
FIGO IIA2	2.67 (1.16~6.13)	0.02	2.63 (0.97~7.14)	0.057
Age	0.97 (0.93~1)	0.088	0.96 (0.91~1)	0.059

Tumor size 2 – 4cm	1.22 (0.6~2.44)	0.584	0.79 (0.34~1.83)	0.581
Tumor size > 4cm	1.32 (0.63~2.78)	0.466	0.44 (0.14~1.38)	0.157
BP YES	0.64 (0.29~1.43)	0.278	0.76 (0.29~1.95)	0.565
DM YES	1.73 (0.66~4.53)	0.266	1.96 (0.64~5.99)	0.236
Neoadjuvant Yes	1.36 (0.74~2.48)	0.32	1.05 (0.47~2.35)	0.911
AFP ≥ 8.78ng/ml	0 (0~Inf)	0.985	0 (0~Inf)	0.99
Ca125 ≥ 35U/ml	4.38 (1.76~10.87)	0.001	2.65 (0.86~8.16)	0.09
Ca153 ≥ 31U/ml	0 (0~Inf)	0.985	0 (0~Inf)	0.99
Ca19-9 ≥ 37U/ml	7.3 (2.14~24.92)	0.001	5.63 (1.36~23.27)	0.017
SCC-Ag ≥ 3.75ng/ml	3.1 (1.67~5.76)	<0.001	4.16 (1.72~10.05)	0.002
LDH	1 (1~1)	0.014	1 (1~1)	0.052
HBG	0.98 (0.96~1)	0.086	1 (0.97~1.03)	0.857
NE%	1.02 (1~1.05)	0.088	1.02 (0.94~1.11)	0.613
WBC	1.03 (0.89~1.19)	0.726	0.87 (0.68~1.12)	0.286
LY%	0.97 (0.94~1)	0.057	0.99 (0.91~1.07)	0.721
PLT	1 (1~1)	0.844	1 (0.99~1)	0.928
HCT	0.94 (0.88~0.99)	0.024	0.96 (0.89~1.05)	0.401

Table S4. Calculation of points and linear predictors.

Characteristics	Points
FIGO IB1	0
FIGO IB2	36.32562343
FIGO IIA1	47.96997781
FIGO IIA2	51.32711645
Age	points = 0 * age ^{3} + 0 * age ^{2} + -2.222222222 * age + 155.555555556
LDH	points = 0.112350636 * LDH + 0
Ca125 < 35U/ml	0
Ca125 ≥ 35U/ml	54.65101822
Ca19-9 < 37U/ml	0
Ca19-9 ≥ 37U/ml	83.04642264
SCC-Ag < 3.75ng/ml	0
SCC-Ag ≥ 3.75ng/ml	54.88529022
Linear.Predictors	LP = 0.019568988 * points + -4.253985012

Table S5. Multivariate and univariate logistic regression for the prediction of LNM based on different risk groups.

Characteristics	Univariate analysis		Multivariate analysis	
	OR(95% CI)	*P* value	OR(95% CI)	*P* value
middle risk group	1.57 (0.35~7.1)	0.56	1.45 (0.31~6.91)	0.638
high risk group	12.19 (2.76~53.85)	0.001	13.95 (2.8~69.42)	0.001
Age	0.97 (0.93~1)	0.088	1 (0.96~1.05)	0.827

Tumor size 2 – 4cm	1.22 (0.6~2.44)	0.584	0.82 (0.36~1.86)	0.641
Tumor size > 4cm	1.32 (0.63~2.78)	0.466	0.49 (0.18~1.37)	0.175
BP YES	0.64 (0.29~1.43)	0.278	0.77 (0.31~1.95)	0.581
DM YES	1.73 (0.66~4.53)	0.266	2.13 (0.68~6.72)	0.195
Neoadjuvant Yes	1.36 (0.74~2.48)	0.32	1.21 (0.54~2.69)	0.64
AFP ≥ 8.78ng/ml	0 (0~Inf)	0.985	0 (0~Inf)	0.99
Ca153 ≥ 31ng/ml	0 (0~Inf)	0.985	0 (0~Inf)	0.989
HBG	0.98 (0.96~1)	0.086	1 (0.98~1.03)	0.753
NE%	1.02 (1~1.05)	0.088	1.02 (0.94~1.1)	0.706
WBC	1.03 (0.89~1.19)	0.726	0.89 (0.69~1.14)	0.347
LY%	0.97 (0.94~1)	0.057	0.98 (0.91~1.06)	0.648
PLT	1 (1~1)	0.844	1 (0.99~1)	0.774
HCT	0.94 (0.88~0.99)	0.024	0.96 (0.89~1.05)	0.381

Table S6. Low-risk patients with LNM.

	the model-development cohort		the validation cohort
Characteristics	Patient 1	Patient 2	Patient 3
2009 FIGO	IB1	IB1	IB1
Tumor size	＞5cm	＜2cm	2 – 4cm
2018 FIGO	IB3	IB1	IB2
DSI	middle 2/3	＜1/3	middle 2/3
LVSI	Present	Present	Absent
PM invasion	Absent	Absent	Absent
Surgical margin	Absent	Absent	Absent
Peritoneal LNs	6	0	0
Peritoneal PLNs	0	0	0
Pelvic PLNs	2	1	1
Pelvic LNs	37	30	51

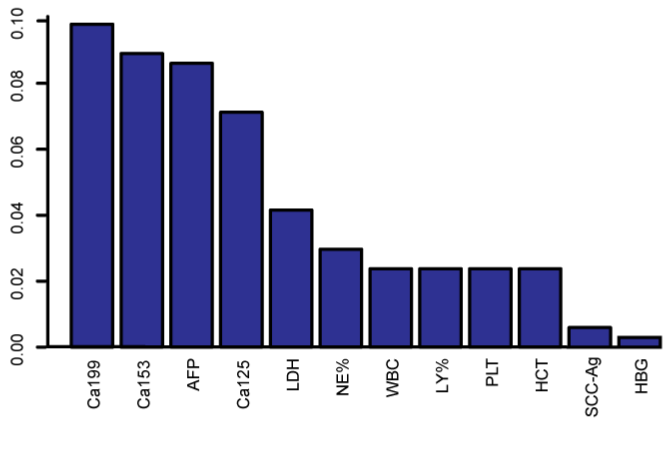

Figure S1. Hematological indicators that were missing from the model-development cohort.

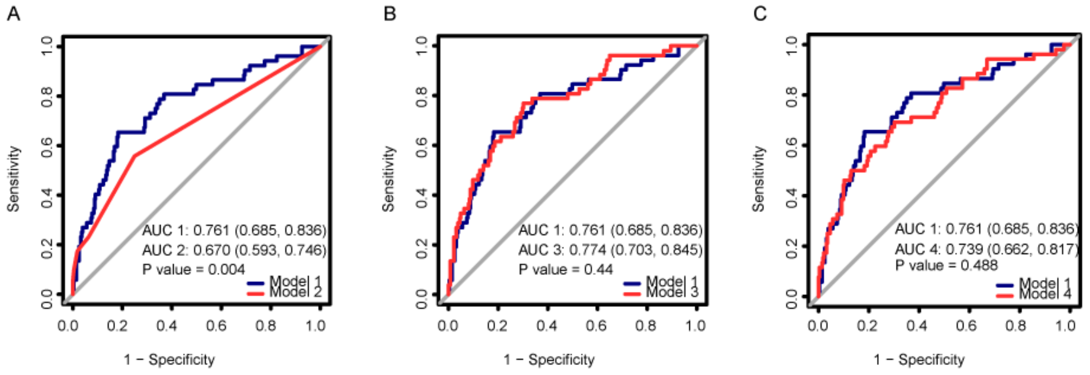

Figure S2. (A) Compare ROC curve and C-index of Model1 and Model2. (B) Compare ROC curve and C-index of Model1 and Model3. (C) Compare ROC curve and C-index of Model1 and Model4

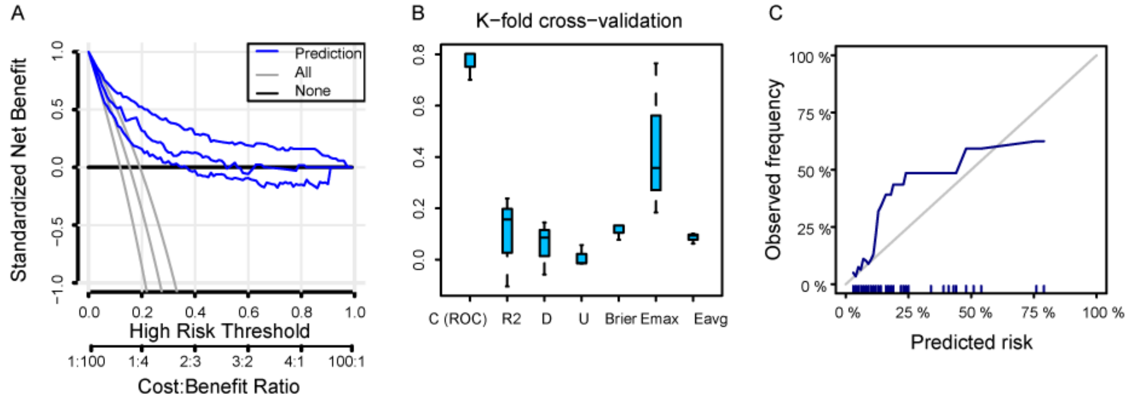

Figure S3. (A) Calibration plots for the model-development cohort. (B) Discrimination and calibration plots for LNM in the internal verification. (C) Calibration plots for the validation cohort.

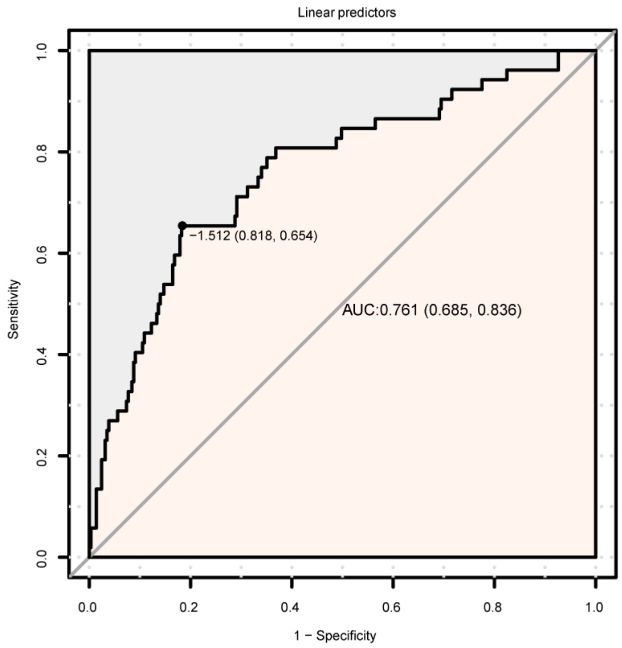

Figure S4. ROC curve of LNM and linear predictors.

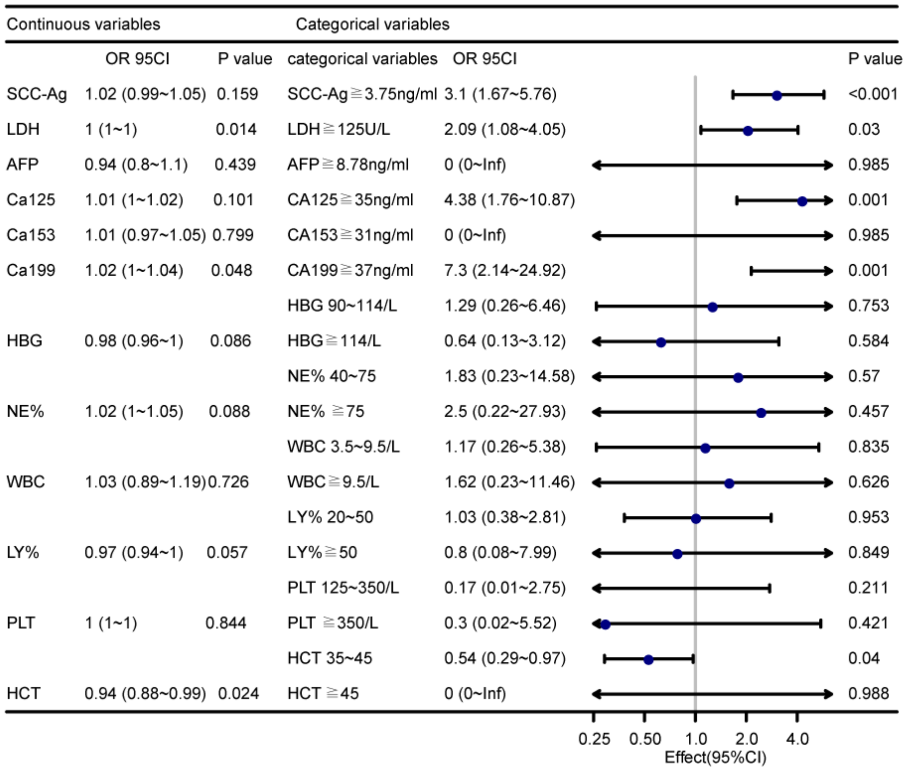

Figure S5. Hematological indicators were considered as both continuous and categorical variables in the univariate analysis to account for all possible relationships with LNM.
